# Studies on the Physical Properties of TiO_2_:Nb/Ag/TiO_2_:Nb and NiO/Ag/NiO Three-Layer Structures on Glass and Plastic Substrates as Transparent Conductive Electrodes for Solar Cells

**DOI:** 10.3390/nano11061416

**Published:** 2021-05-27

**Authors:** Laura Hrostea, Petru Lisnic, Romain Mallet, Liviu Leontie, Mihaela Girtan

**Affiliations:** 1 Research Center on Advanced Materials and Technologies, Science Department, Institute of Interdisciplinary Research, Alexandru Ioan Cuza University of Iasi, Bldv. Carol I, No. 11, 700506 Iasi, Romania; laura.hrostea@uaic.ro; 2 Faculty of Physics, Alexandru Ioan Cuza University of Iasi, Bldv. Carol I, No. 11, 700506 Iasi, Romania; petru.lisnic@yahoo.com (P.L.); lleontie@uaic.ro (L.L.); 3 SCIAM, SFR ICAT, Université d’Angers, 4 Rue Larrey, CEDEX 09, 49033 Angers, France; romain.mallet@univ-angers.fr; 4 Photonics Laboratory, (LPhiA) E.A. 4464, SFR Matrix, Faculté des Sciences, Université d’Angers, 2 Bd Lavoisier, 49000 Angers, France

**Keywords:** oxide/metal/oxide, OMO, DMD, ellipsometry, transparent conductive electrodes, plastic substrates, organic solar cells, perovskite solar cells, contact angle, wettability

## Abstract

In this paper, the physical properties of a new series of multilayer structures of oxide/metal/oxide type deposited on glass and plastic substrates were studied in the context of their use as transparent conductive layers for solar cells. The optical properties of TiO_2_/Ag/TiO_2_, TiO_2_:Nb/Ag/TiO_2_:Nb and NiO/Ag/NiO tri-layers were investigated by spectrophotometry and ellipsometry. Optimized ellipsometric modeling was employed in order to correlate the optical and electrical properties with the ones obtained by direct measurements. The wetting surface properties of single layers (TiO_2_, TiO_2_:Nb and NiO) and tri-layers (TiO_2_/Ag/TiO_2_ TiO_2_:Nb/Ag/TiO_2_:Nb and NiO/Ag/NiO) were also studied and good correlations were obtained with their morphological properties.

## 1. Introduction

In the class of transparent conducting electrodes, there are few highly-doped oxides that are typically used as single layers of about 100 to 200 nm for electronics and solar cell applications [1]. Among these, the most well-known is Sn-doped In_2_O_3_ (ITO–indium tin oxide). Due to its intensive use and extremely limited resources on Earth, indium is one of the most economically important critical raw materials [2]. Hence, alternative solutions for ITO have been intensively looked for. A lot of studies have been done on Al, In and Ga-doped ZnO (AZO, IZO and GZO) thin films, and on F-doped SnO_2_ (FTO) [3,4,5,6,7,8,9,10,11,12,13]. Besides, in the last few years, a new class of electrodes including ITO/Au/ITO, ITO/Ag/ITO, ZnO/Au/ZnO, AZO/Au/AZO and Bi_2_O_3_/Au/Bi_2_O_3_ [14,15,16,17] was developed on plastic substrates for OPV applications [18,19]. A lot of studies were also done on using TiO_2_/Ag/TiO_2_ as an electrode, especially for DSSC applications and perovskite solar cells, due to such electrodes’ energy conversion efficiency [20,21,22,23,24,25,26,27,28,29,30]. These oxide/metal/oxide (O/M/O) electrodes have many advantages, due to their suitability for deposition on flexible substrates. Of their favorable mechanical properties, the metallic layer’s ductility is notable. The necessary quantity of oxide materials is generally reduced by two or three times; hence, the total electrode film’s thickness can be reduced. The oxide layers act as protective coatings against the oxidation and mechanical degradation of the metallic interlayer film. For solar cell applications, the surface film’s properties positively influence the values of the extraction potential. 

The novelty of this study consists in its comprehensive analysis of a new class of oxide/metal/oxide electrodes, including TiO_2_:Nb/Ag/TiO_2_:Nb and NiO_x_/Ag/NiO_x_ (for simpler reading, we use the notation NiO/Ag/NiO for the last structure), which were deposited on plastic and glass substrates by sputtering from metallic targets. 

Indeed, very few studies have been done on TiO_2_:Nb/Ag/TiO_2_:Nb [31,32] and NiO/Ag/NiO [33,34,35]. Recently, it was proved that the NiO/Ag/NiO antireflective multilayer electrodes used as top cathodes [33], bottom electrodes for CH_3_NH_3_PBI_3_ perovskite solar cells [34], or bottom electrodes for PBDTTT-C:PCBM organic solar cells, have improved efficiency compared to the industry standard [35]. To further this important progress for organic and perovskite solar cells, the purpose of this paper is to give a complete and comparative overview of the physical properties of three of these new electrodes: TiO_2_/Ag/TiO_2_, TiO_2_:Nb/Ag/TiO_2_:Nb and NiO/Ag/NiO.

## 2. Materials and Methods

The study involved the preparation and analysis of three sets of samples, including single layers and three-layer oxide/metal/oxide structures deposited on plastic (HIFIPMX739 PET) and glass substrates. Thin oxide films and the metallic interlayer film were deposited by DC magnetron sputtering in reactive and argon atmospheres, respectively, using different metallic targets. The deposition was performed at room temperature in a vertical target–substrate configuration. The deposition parameters were the same regardless of the structures with which the layers were involved, which are mentioned in Table 1. They were chosen taking in account the optimal values in order to obtain simultaneously good optical and electrical properties. 

The morphological properties were analyzed by electron microscopy and atomic force microscopy using a CP-R, Veeco thermo-microscope (CSInstruments, Les Ulis, France) and a JEOL JSM 6301F Electronic Microscope (JEOL, Croissy-sur-Seine, France). The wetting properties were studied via contact angle measurements performed at room temperature using distillated water droplets of equal volumes (3 µL). The optical properties were investigated on both single oxide layers and oxide/metal/oxide layers, using several techniques. Information regarding the transmission and reflection spectra was recorded using a double beam UV/VIS S9000 (Labomoderne, Gennevilliers, France) spectrophotometer and an AvaSpec-3648 Avantes optical fiber spectrophotometer (Avantes, Apeldoorn, The Netherlands), respectively. The optical properties were studied in a 300–1100 nm wavelength range. For instance, the amplitude (*ψ*) and phase difference (Δ) spectra were registered by spectroscopic ellipsometry using an UVISEL NIR Horiba Jobin Yvon ellipsometer (Horiba Jobin Yvon, Longjumeau, France) equipped with a 75 W high discharge Xe lamp. The chosen configurations for the modulator (M), analyzer (A) and polarizer (P) positions were: M = 0° and A = 45°; the incidence angle was AOI = 70°. The experimental data were fitted by modelling using the Delta Psi 2 software from Horiba Jobin Yvon (Horiba Jobin Yvon, Longjumeau, France). The optimization of the models was done by following the procedure described in [17]. The electrical conductivity measurements were done using four-point method in planar geometry at room temperature using a Keithley 2600 source Metter (RS Components Ltd., Northants, UK), by measuring the total (sheet) resistance of the multilayer structure. The distance between the probe tips was 0.635 mm. The electrical conductivity was calculated using the estimated value of thickness obtained by ellipsometry for the three-layer structure.

## 3. Results and Discussion

**Figure 1:**
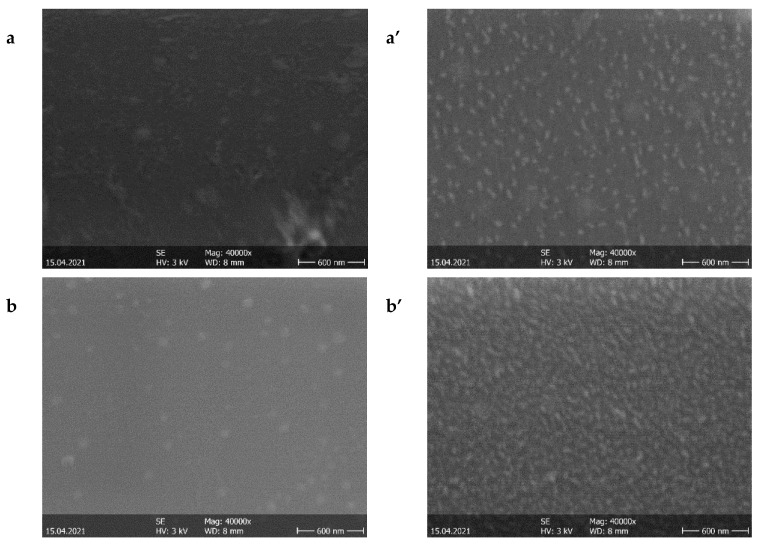

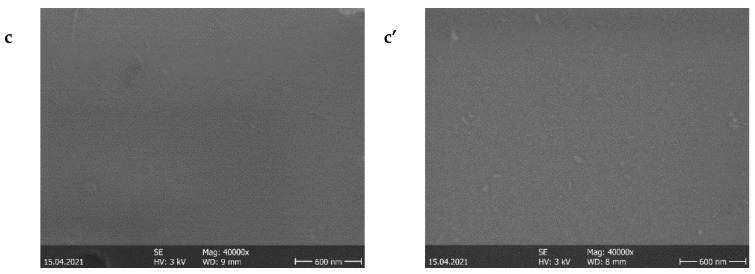
SEM micrographs of single oxide (bottom) layers: (**a**) TiO_2_, (**b**) TiO_2_:Nb and (**c**) NiO; and SEM micrographs of the top oxide layers of oxide/Ag/oxide multilayer structures: (**a’**) TiO_2_, (**b’**) TiO_2_:Nb and (**c’**) NiO.

Figure 1a–c depicts the SEM micrographs of the bottom oxide films prior to the deposition of subsequent layers, and Figure 1a–c depicts the top of the second oxide layer of the three-layer structures (oxide/metal/oxide). 

**Figure 2:**
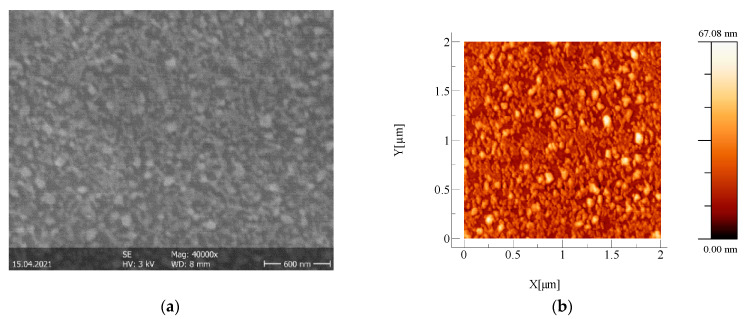
(**a**). SEM image of the Ag interlayer and (**b**) AFM image of the Ag interlayer.

Figure 2 illustrates the SEM and AFM images of the silver interlayer. The AFM analysis was done both for the surfaces of the oxide single layers deposited on glass (not shown here) and for the top oxide layers of the oxide/metal/oxide multilayer structures deposited on glass. The root mean square (RMS) and average (RA) roughness values of these layers are given in Table 2. 

Regarding the topography and morphological properties, the SEM micrographs show that the silver layer influenced the surface morphologies of the top surfaces of the O/M/O structures with TiO_2_ and TiO_2_:Nb, but this influence was smaller for the NiO-based structure. This can be explained, on the one hand, by the fact that the thickness of the NiO second layer was greater than those of the other two oxides (see Table 3), and on the other hand, by the fact that NiO’s roughness value was lower than those of TiO_2_ and TiO_2_:Nb layers. 

**Figure 3:**
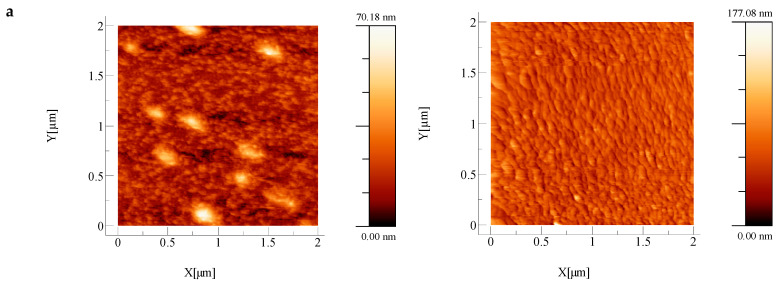

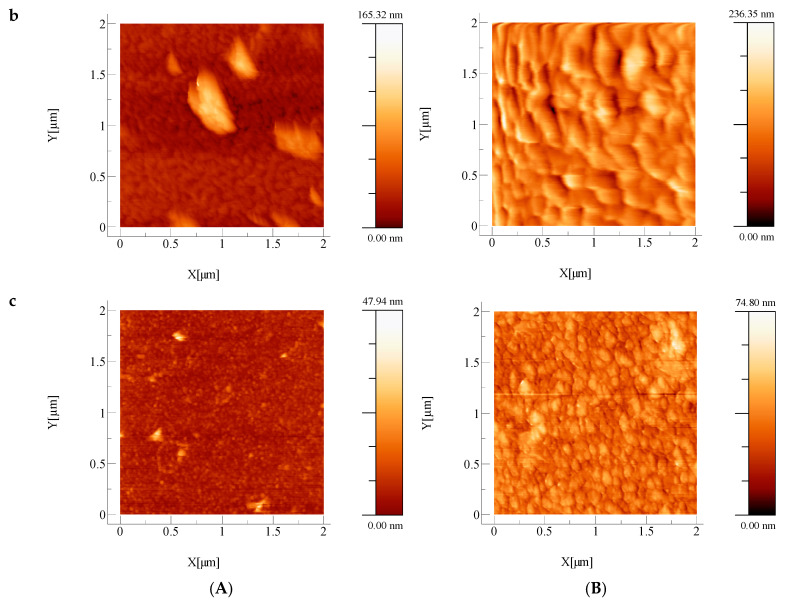
AFM analyses of top surfaces of oxide/Ag/oxide structures deposited on glass (**A**) and on PET (**B**): (**a**) TiO_2_/Ag/TiO_2_, (**b**) TiO_2_:Nb/Ag/TiO_2_:Nb and (**c**) NiO/Ag/NiO.

**Figure 4:**
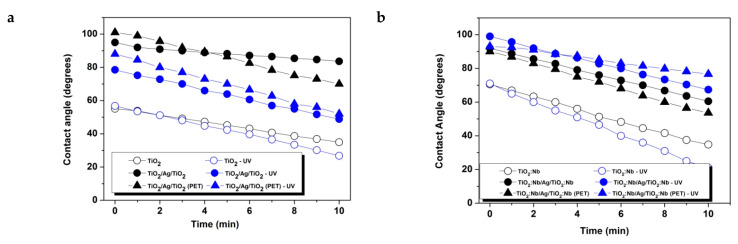

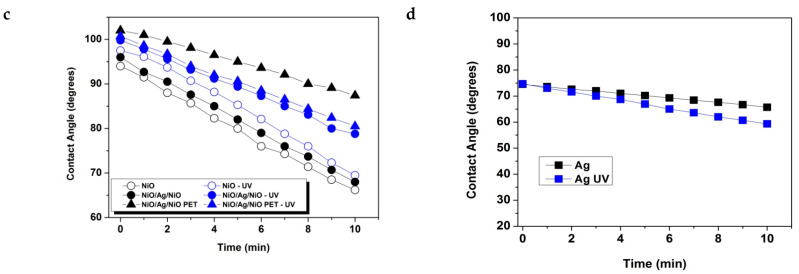
Contact angle measurements with and without UV irradiation for single and three-layer samples: (**a**) TiO_2_; (**b**) TiO_2_:Nb; (**c**) Nio; (**d**) Ag interlayer.

Figure 3 reproduces the AFM images obtained by scanning the top surfaces of the oxide/metal/oxide layers deposited on glass and on plastic substrates. As was the case for films of ITO/Metal/ITO, AZO/Metal/AZO and ZnO/Metal/ZnO studied previously [15], the films deposited on plastic substrates were rougher than the films deposited on glass substrates (see Table 2). Since the morphology in this context is closely related to wettability expressed in terms of contact angles, we measured such contact angles, and the results are given in Table 2. As one can see, the existence of the metallic interlayer increased the contact angle of the oxide surface in every case. All surfaces were also sensitive to UV exposure. The changes of the contact angles as a function of time with and without exposure to UV irradiation by using a 254 UV-C a 1 × 8W EF180C 1180 mW/cm^2^ lamp, are given in Figure 4. For TiO_2_ films and TiO_2_/Ag/TiO_2_ films on glass and plastic substrates, the contact angles decreased after exposure to UV, indicating that the surfaces became more hydrophilic. This is in agreement with the classical behavior of TiO_2_ films [36]. For TiO_2_:Nb/Ag/TiO_2_:Nb and NiO/Ag/NiO, the influence of UV radiation was quite slight.

Refractive index and film thickness were determined by spectroscopic ellipsometry. Thickness for single layers and the individual thicknesses in multilayer structures were determined by fitting the experimental ellipsometric spectra with those which resulted from theoretical models. For the numerical simulations and modeling, we used the Delta Psi2 software from Horiba Jobin Yvon. The global refractive indices of structures were measured and modelled using the following dispersion formulas: the new amorphous dispersion formula for TiO_2_ and TiO_2_:Nb; the Tauc–Lorentz formula for NiO; and the Drude and Tauc–Lorentz formulae for Ag. 

**Figure 5:**
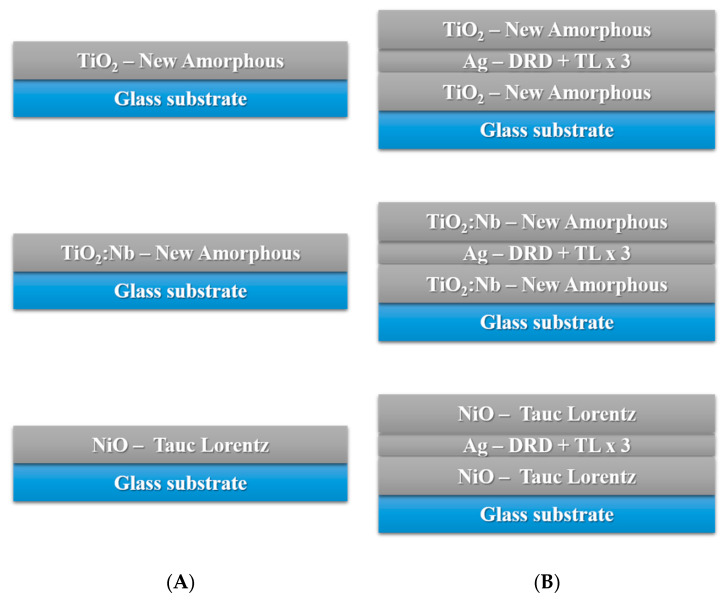
Ellipsometric models for single layers (**A**) and O/M/O three-layer structures (**B**).

Figure 5 illustrates the optical models used for the theoretical calculations and simulations for single layers (A) and O/M/O structures (B).

The films’ thickness values obtained after the optimization of the models as described in [17], are given in Table 3.

**Figure 6:**
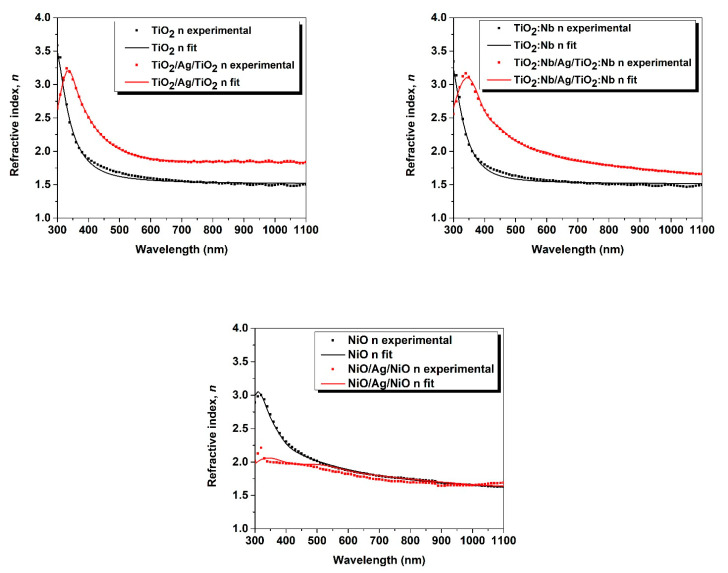
Ellipsometry experimental data (dotted lines) fitted by theoretically calculated curves (continuous lines) from the ellipsometric models, for single and three-layer samples.

In Figure 6, the experimental ellipsometric data for the global refractive index are represented as dotted lines, and the fitting curves as continuous lines. The optimal thickness of the silver interlayer, realized by ellipsometric measurements (Table 3) is of 8 nm [20]. This thickness is the lowest limit because, for lower values, the film does not completely cover the substrate, and islands of Ag might appear which are not interconnected, making the resulting layer not conductive. By increasing the thickness, the metallic interlayer is certainly conductive, but the transparency of the O/M/O electrode is reduced.

As for single-layer films deposited by a sol–gel method—or in this case for films deposited by reactive sputtering—the refractive indices of thin Nb-doped TiO_2_ films are smaller than those of an undoped TiO_2_ thin films [37]. For the multilayer structures TiO_2_/Ag/TiO_2_ and TiO_2_:Nb/Ag/TiO_2_:Nb, the refractive indices were higher than those of single layers. On the contrary, the refractive index of NiO/Ag/NiO was smaller than the refractive index of the NiO single layer. These optical properties are important, since these films are used as electrodes for solar cells and optoelectronic devices.

**Figure 7:**
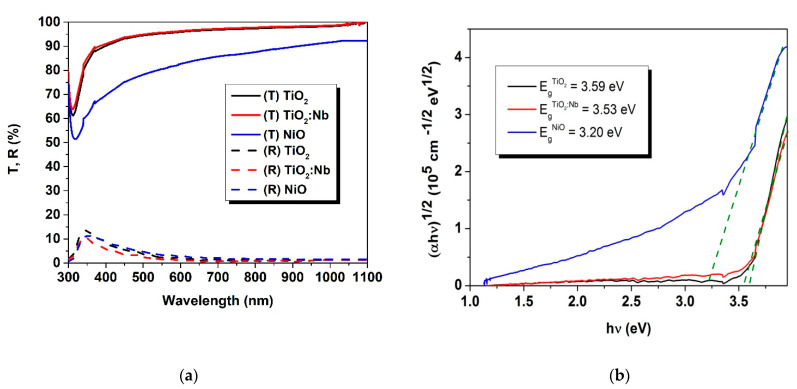
(**a**) Transmittance and reflectance spectra for the single-layer coatings; (**b**) energy band gap calculation using spectrophotometry data.

The transmittance and reflectance spectra for single layers, obtained by spectrophotometry, are given in Figure 7a. From these spectra, the optical energy band gaps were calculated using the Tauc plots (Figure 7b) for indirect optical transitions. The calculated band gap values were compared with the results from the ellipsometric modelling and those given in literature (see Table 4). We can report a satisfactory correlation between the values obtained by different methods, and satisfactory correlations with those reported by others authors—this being the second verification of the validity of the ellipsometric optical models. 

**Figure 8:**
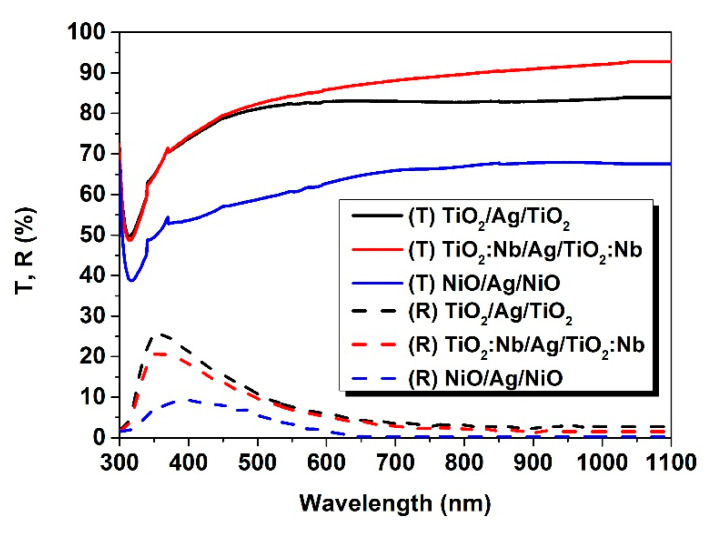
Transmittance and reflectance spectra for the O/M/O structures by spectrophotometry.

Similarly to Figure 7, Figure 8 gives the transmittance and reflectance spectra for O/M/O layers. Due to the increased thicknesses of these structures, the transmittance was 10% lower. 

Due to the presence of the Ag interlayer, the reflectance increased consistently by 10%, except for the NiO/Ag/NiO three-layer structure, for which the optical features of silver were reduced by the thicker NiO top layer.

The electrical resistivity values determined from direct measurements and also from ellipsometric calculations were roughly 7 × 10^−3^ Ω∙cm for TiO_2_/Ag/TiO_2_, 1 × 10^−4^ Ω∙cm for TiO_2_:Nb/Ag/TiO_2_:Nb and 2 × 10^−4^ Ω∙cm for NiO/Ag/NiO, and are in line with the values obtained by other authors [32,33,35,40] for films deposited on oxide targets. The correlation between the electrical measurements and the ellipsometric simulations is demonstrated by equivalent values of plasma frequency. Therefore, taking into account Drude’s model describing the kinetic theory of electrons in metal, plasma frequency is defined as follows [39]:

(1) $$\omega_{p}=\sqrt{\frac{4\pi \sigma }{\varepsilon_{0}\varepsilon_{\infty }\langle \tau \rangle }}$$

where *σ* represents the electrical conductivity; $\varepsilon_{0}$ is the vacuum permittivity ($\varepsilon_{0}=8.85\times 10^{-12}F/m)-\varepsilon_{\infty }=1$ generally, according to the Lorentz dispersion model, on which Drude’s model is based; and 〈τ〉 is the relaxation time of electrons (for Ag electrons, $\langle \tau \rangle ≅4\times 10^{-14}\;s$ [40,41]). 

The resulting values of plasma frequency based on electrical conductivity (from direct measurements) are compared with the values of plasma frequency released in the ellipsometric simulations of samples in Table 5.

The differences in the values of plasma frequency calculated from electrical measurements and from ellipsometric modelling are within reasonable limits when taking into account the fact that the spectroscopic ellipsometry technique is based on reflections at one point (local measurements) and also taking into account the limits in the accuracy of the models.

By analyzing all these data, we can conclude that TiO_2_/Ag/TiO_2_, TiO_2_:Nb/Ag/TiO_2_:Nb and NiO/Ag/NiO have quite similar optical and electrical properties. However, higher values of transparency and electrical conductivity were obtained for TiO_2_:Nb/Ag/TiO_2_:Nb. The NiO/Ag/NiO three-layer electrodes could be slightly improved by reducing the oxide layer’s thickness. The main advantage of NiO/Ag/NiO electrodes is the fact that the refractive index is lower than those of TiO_2_/Ag/TiO_2_ and TiO_2_:Nb/Ag/TiO_2_:Nb. Using ellipsometry, which is a powerful tool, the established optimal models will be used in a future work to simulate the properties of the new optimized structure. 

## 4. Conclusions

We presented a comparative study regarding the physical properties of oxide/metal/oxide three-layer structures which are promising alternatives to ITO electrodes in the photovoltaic field. The oxide layers (TiO_2_, TiO_2_:Nb, NiO) and the metallic interlayer (Ag) were laid by successive DC sputtering deposition on glass and plastic substrates. The performances of this type of electrode architecture were presented from optical and electrical points of view, and we also described the morphological features. The presence of Ag as an interlayer influences the three-layer structure. Firstly, the transmittance shows a decrease of 10%, and the reflectance an increase of 10%, the latter depending on the oxide layer’s thickness. Secondly, the roughness of such a structure is directly dependent on the substrate roughness, and it too is influenced by the silver’s morphological properties. Thirdly, from an electrical point of view, in terms of electrical resistivity (~10^−3^ Ω∙cm), these O/M/O structures presented huge potential for photovoltaic applications as transparent conductive electrodes. The ellipsometry optical models were validated by combining different direct measurements. These ellipsometric models can be now used to simulate the properties of new optimized structures.

## Figures and Tables

**Table 1:** Deposition conditions for the samples.

Layer	Atmosphere Conditions	Target–Substrate Distance (cm)	Deposition Current (mA)	Pressure (10^−3^ mbar)	Deposition Time	Target Composition (wt%)
TiO_2_	Reactive atm.	7	100	9	4 min	Ti 100%
Ag	Argon atm.	7	20	9	18 s	Ag 100%
TiO_2_	Reactive atm.	7	100	9	4 min	Ti 100%
TiO_2_:Nb	Reactive atm.	7	100	9	4 min	Ti 94% Nb 6%
Ag	Argon atm.	7	20	9	18 s	Ag 100%
TiO_2_:Nb	Reactive atm.	7	100	9	4 min	Ti 94% Nb 6%
NiO	Reactive atm.	7	100	10	4 min	Ni 100%
Ag	Argon atm.	7	20	10	18 s	Ag 100%
NiO	Reactive atm.	7	100	10	4 min	Ni 100%

**Table 2:** A summary of RMS and RA roughness values, and contact angle (CA) values of the single oxide layers and the top oxide layers of the multilayer structures (O/M/O) deposited on glass and PET substrates.

Sample	RMS (nm)	RA (nm)	Without UV Light		With UV Light	
			CA (deg) t = 0′	CA (deg) t = 10′	CA (deg) t = 0′	CA (deg) t = 10′
TiO_2_	6.4	4.5	55	35	57	27
TiO_2_/Ag/TiO_2_	8.2	5.9	95	83	79	49
TiO_2_/Ag/TiO_2_ (on PET)	13.1	10.3	101	70	88	52
TiO_2_:Nb	7.8	4.2	70	35	71	21
TiO_2_:Nb/Ag/TiO_2_:Nb	16	9.9	91	60	99	67
TiO_2_:Nb/Ag/TiO_2_:Nb (on PET)	26.5	20.7	90	54	93	77
NiO	3.4	2.0	93	65	98	70
NiO/Ag/NiO	2.6	1.8	96	68	100	79
NiO/Ag/NiO (on PET)	6.8	5.4	102	87	101	81
Ag	8.7	6.9	75	66	75	60

**Table 3:** Thicknesses obtained from ellipsometry simulations.

Sample		Thickness (nm)		Χ^2^
**TiO_2_**		28 ± 1		4.55
**TiO_2_:Nb**		28 ± 1		4.90
**NiO**		63 ± 1		3.41
	**Oxide** **bottom layer**	**Ag**	**Oxide** **top layer**	
**TiO_2_/Ag/TiO_2_**	24 ± 1	8 ± 1	37 ± 1	0.85
**TiO_2_:Nb/Ag/TiO_2_:Nb**	34 ± 1	8 ± 1	42 ± 2	0.55
**NiO/Ag/NiO**	42 ± 9	8 ± 1	72 ± 9	7.9

**Table 4:** Energy band gap values from spectrophotometric and ellipsometric measurements.

Sample	E_g_ by Ellipsometry (eV)	E_g_ by Spectrophotometry (eV)	E_g_ by Literature (eV)	Reference
TiO_2_	3.36	3.59	3.28–3.32	[36]
TiO_2_:Nb	3.18	3.53	3.25–3.58	[37]
NiO	3.76	3.20	3.60–4.00	[38,39]

**Table 5:** A comparison between plasma frequency values obtained from direct electrical measurements and from ellipsometric simulations.

Sample	*ω_p_* (s^−1^) Using Formula (1) and the Direct Measured Values of σ	*ω_p_* (s^−1^) From Ellipsometric Modeling
TiO_2_/Ag/TiO_2_	$0.7\times 10^{15}$	$\left(4.9\pm 1.5\right)\times 10^{15}$
TiO_2_:Nb/Ag/TiO_2_:Nb	$5.6\times 10^{15}$	$\left(67.0\pm 17.5\right)\times 10^{15}$
NiO/Ag/NiO	$4.2\times 10^{15}$	$\left(12.6\pm 1.3\right)\times 10^{15}$
